# Metformin Use Is Associated with Reduced Incidence and Improved Survival of Endometrial Cancer: A Meta-Analysis

**DOI:** 10.1155/2017/5905384

**Published:** 2017-03-20

**Authors:** Yun-Liang Tang, Ling-Yan Zhu, Yu Li, Jiao Yu, Jiao Wang, Xiang-Xia Zeng, Kai-Xiang Hu, Jian-Ying Liu, Ji-Xiong Xu

**Affiliations:** ^1^Department of Endocrinology and Metabolism, First Affiliated Hospital of Nanchang University, Nanchang, Jiangxi 330006, China; ^2^Department of Pharmacy, Pingxiang Maternal and Child Health Care Hospital, Pingxiang, Jiangxi 337000, China

## Abstract

Studies have suggested that metformin can potentially decrease the incidence of cancer and improve survival outcomes. However, the association between metformin use and the incidence and survival of endometrial cancer (EC) remains controversial. So, a meta-analysis was performed. An electronic search was conducted using PubMed, EMBASE, and Web of Science. The outcome measures were relative risks (RRs) or hazard ratios (HRs) with 95% confidence intervals (CIs) comparing the EC incidence and survival in patients treated with and without metformin. Eleven studies involving 766,926 participants were included in this study. In the pooled analysis of five studies which evaluated the association of metformin use with the incidence of EC, we found that metformin use was associated with a 13% reduction in EC risk among patients with diabetes (RR = 0.87, 95% CI: 0.80–0.95; *p* = 0.006). In the pooled analysis of six retrospective cohort studies evaluating the effect of metformin on the survival of EC patients, we found that, relative to nonuse, metformin use significantly improved the survival of EC patients (HR = 0.63, 95% CI: 0.45–0.87; *p* = 0.006). This study showed that metformin use was significantly associated with a decreased incidence of EC in diabetes and a favorable survival outcome of EC patients.

## 1. Introduction

Endometrial cancer (EC) is the most commonly occurring gynecologic malignant tumor of the female reproductive system, and its incidence is increasing worldwide [1, 2]. Both diabetes and obesity are risk factors which promote the development and progression of EC [3]. Primary surgical treatment is the mainstay of therapy, including total hysterectomy and bilateral salpingooophorectomy [4]. Unfortunately, many patients diagnosed with local or advanced EC will still suffer from recurrence or die of this disease, although some of them had been cured. Thus, many efforts are needed to reduce the incidence of EC as well as research to identify novel therapeutic targets with the aim of improving the disease survival.

Metformin, an oral biguanide, one of the most commonly prescribed antidiabetic medications [5], may have antineoplastic properties. Recent epidemiological studies have reported potential beneficial effects of metformin on gynecological cancers. Metformin use significantly decreased the risk or improved some survival outcomes among patients with cervical, breast, and ovarian cancer [6–8]. Moreover, several meta-analyses have shown that metformin use decreased the incidence and improved the survival of a wide range of malignant tumors, such as liver cancer, lung cancer, prostate cancer, and colorectal cancer [9–15]. Although the exact mechanism is still not fully understood, studies in vivo and vitro have shown that metformin likely exerts its antitumorigenic effects directly or through other downstream targets to inhibit the growth and proliferation of tumor cells. Direct mechanisms may be the activation of 5-AMP-activated protein kinase (AMPK), which results in the inactivation of mammalian target of rapamycin (mTOR) [16]. Indirect mechanisms may be the inhibition of liver gluconeogenesis, resulting in a decrease in insulin levels and hyperglycemia [17].

Thus far, several clinical researches have investigated the effect of metformin on the incidence of EC in diabetic patients. However, it is still uncertain whether the use of metformin could decrease the incidence of EC owing to the contradictory results of these studies. Tseng reported that the use of metformin in women with diabetes was associated with an overall significantly lower risk of EC [18]. However, Franchi et al.'s study indicated that metformin did not meaningfully affect the risk of EC [19]. Moreover, studies have also investigated the association between metformin use and survival of EC patients. However, whether metformin use could generate better clinical outcomes in EC patients also remains unclear. Ko et al. reported that the nonmetformin users had 2.3-fold increased risk of death when compared with the metformin users after adjusting for age, stage, grade, histology, and adjuvant treatment [20]. Conversely, Al Hilli et al. reported that overall survival was similar between the metformin users and nonusers of EC patients after adjusting for confounding covariates [21]. Based on these studies, the association between metformin use and the incidence and survival of EC is still uncertain; thus, a meta-analysis is needed to confirm the effects of metformin.

## 2. Materials and Methods

### 2.1. Literature Search

A comprehensive literature search was performed to identify all potentially relevant articles using the PubMed, EMBASE, and Web of Science database from their inception to 20 October 2016. The search was restricted to the articles published in English. We developed a search strategy using the following terms: “metformin” or “biguanide” and “endometrial cancer” or “endometrial carcinoma”. Additionally, we screened bibliographies of the selected original studies and review articles to identify any other relevant studies that were not captured through the initial database searches.

### 2.2. Eligibility Criteria

Studies were included if they met the following criteria: (a) randomized controlled clinical trials, case-control studies, nested case-control studies, and cohort studies; (b) studies evaluating the association between metformin use and incidence of EC in diabetes or those evaluating the effect of metformin on survival of EC patients; (c) studies reporting relative risks (RRs) or hazard ratios (HRs) with corresponding 95% confidence intervals (CIs) or providing sufficient data to calculate these values. If more than one paper was based on the same study, we included the one which provided the most abundant information or the one containing the largest number of cases. Studies were excluded if they were publications from letters, editorials, reviews, cell line studies, animal studies, and studies without controls.

### 2.3. Data Extraction Quality Assessment

Two authors performed the data extraction independently. For the eligibility studies, the collected information included first author, year of publication, region, study design, data source, sample size, time period, RR (95% CI) or HR (95% CI), and variables controlled for matching or used in multivariable models. If several estimates were reported in the same article, the most fully adjusted estimate was selected. Disagreements were resolved by discussion between two reviewers. When required, disagreements were resolved by consultation with a third reviewer (Xu).

To assess the quality of included studies, the Newcastle-Ottawa Scale (NOS) was applied in this meta-analysis [29]. We evaluated the included studies based on selection of participants, comparability of participants, and ascertainment of outcomes and then scored the methodological quality. Quality assessment was independently conducted by two authors, with a third party (Xu) assessment when necessary.

### 2.4. Statistical Analyses

To assess the association between metformin use and the incidence and survival of EC, summary RRs and HRs with their corresponding 95% CIs were calculated through meta-analysis. Heterogeneity analysis was performed by Cochran *Q* statistic and *I*^2^ statistic. Statistical significance for heterogeneity was considered if *p* < 0.05 or *I*^2^ > 50%. The fixed-effects model was applied when *p* > 0.05 and *I*^2^ < 50%, while the random-effects model was chosen when *p* < 0.05 or *I*^2^ > 50%. To identify the sources of heterogeneity, we performed subgroup analyses. Additionally, we conducted sensitivity analyses by removing one study each time and recalculating pooled effects. Potential publications bias (considered present if *p* ≤ 0.1) was assessed by conducting statistical tests for funnel plot asymmetry as well as Egger's test and Begg's test. All statistical tests were conducted using Stata software (Version 12; StataCorp, College Station, TX, USA).

## 3. Results

### 3.1. Study Characteristics

**Figure 1:**
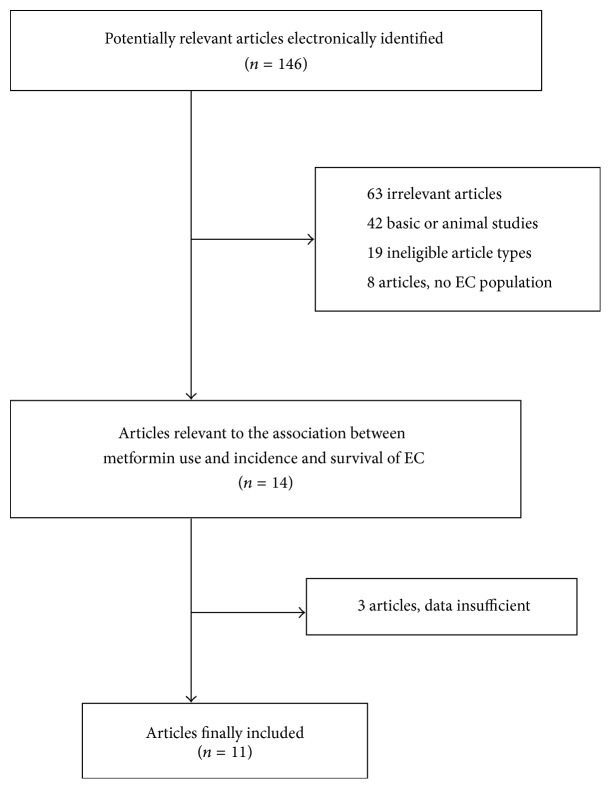
Flow diagram of study selection.

The participant flow diagram for the study inclusion in the metaanalysis is shown in Figure 1. Finally, 11 relevant studies were retrieved, comprising a total of approximately 766,926 participants. Five studies referred to the incidence of EC with a total of 764,810 participants [18, 19, 22–24]. Six studies investigated the survival of EC with 2,116 patients [20, 21, 25–28]. Information on region, data source, time period, sample size, and adjustment variables is presented in Tables 1 and 2. Nine studies were designed as retrospective cohort studies, one was nested case-control study, and one was case-control study. Five studies were based in Poland, Austria, Taiwan, UK, and Italy, respectively. Six studies were based in the USA. Of the included studies, adjusted multivariate analyses for the effect of metformin were performed in 9 studies, and unadjusted univariate analyses were performed in 2 studies. The NOS score of the selected studies ranged from 6 to 8 stars on the scale, which suggested moderate to high quality.

### 3.2. Quantitative Synthesis

#### 3.2.1. Metformin Use and Incidence of Endometrial Cancer

**Figure 2:**
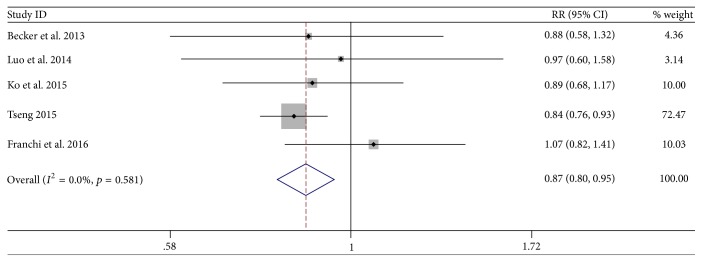
Forest plot of the association between metformin use and incidence of endometrial cancer.

Based on the combined results of the five studies, compared with the reference groups, metformin use was significantly associated with a decreased incidence of EC in diabetes (RR = 0.87, 95% CI: 0.80–0.95; *p* = 0.006; Figure 2). Moreover, there was no obvious between-study heterogeneity in this meta-analysis of five studies in total (*I*^2^ = 0.0%). Stratification according to study design showed that metformin use was significantly associated with a decreased incidence of EC for retrospective studies (RR = 0.85, 95% CI: 0.78–0.93; *p* = 0.001; *I*^2^ = 0.0%), but not for case-control studies (RR = 0.88, 95% CI: 0.58–1.32, *p* = 0.542) or nested case-control studies (RR = 1.07, 95% CI: 0.82–1.41, *p* = 0.625).

#### 3.2.2. Metformin Use and Survival of Endometrial Cancer

**Figure 3:**
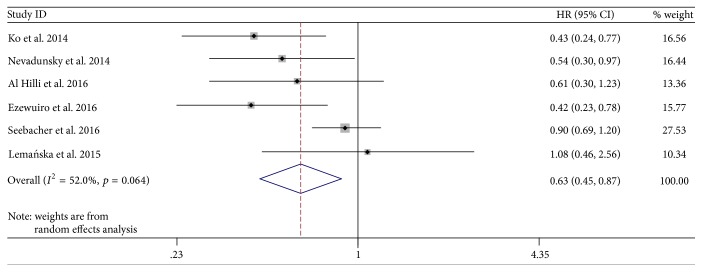
Forest plot of the association between metformin use and survival of endometrial cancer.

Compared with no metformin use, metformin use was associated with survival improvement of EC patients (HR = 0.63, 95% CI: 0.45–0.87; *p* = 0.006; Figure 3). Figure 3 also shows the HRs (95% CI) for each individual study comparing the group of metformin use with the reference group (no metformin use). There was obvious between-study heterogeneity in this meta-analysis of six studies in total (*I*^2^ = 52.0%). In the stratified analyses by adjustment, pooled analysis of four studies with adjustment of variables showed the same effect (HR = 0.49, 95% CI: 0.36–0.46; *p* = 0.000) with low heterogeneity (*I*^2^ = 0.0%). However, two studies without adjusting variables showed that metformin use was not associated with the overall survival of patients with EC (HR = 0.92, 95% CI: 0.70–1.19; *p* = 0.512; *I*^2^ = 0.0%). Stratified analyses by reference group showed that the beneficial effect of metformin use was stable (HR = 0.47, 95% CI: 0.33–0.67; *p* = 0.000; *I*^2^ = 0.0%), when the controlled nonmetformin users were restricted to EC patients with diabetes. However, when compared with nonmetformin users with and without diabetes, the beneficial effect on survival lost significance (HR = 0.84, 95% CI: 0.66–1.07; *p* = 0.149; *I*^2^ = 27.2%).

#### 3.2.3. Sensitivity Analysis

Sensitivity analyses were conducted to verify the effect of each study on the overall estimate by omitting one study at a time and calculating the combined results for the remaining studies. Finally, we found that no individual study significantly affected the pooled RR and HR.

#### 3.2.4. Publication Bias

**Figure 4:**
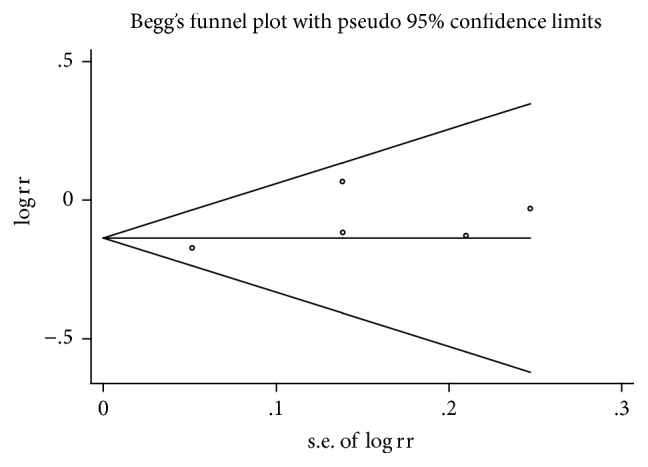
Begg's funnel plots for publication bias test on the association of metformin use with the incidence of endometrial cancer (*p* = 0.231 for Egger's test and *p* = 0.707 for Begg's test).

**Figure 5:**
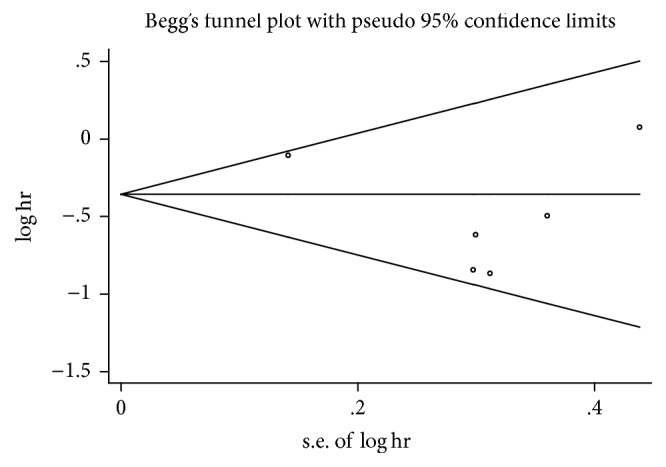
Begg's funnel plots for publication bias test on the association of metformin use with the survival of endometrial cancer (*p* = 0.220 for Egger's test and *p* = 0.806 for Begg's test).

To assess the possibility of publication bias among the studies, funnel plots were generated (Figures 4 and 5). Finally, no evident asymmetry of the funnel plot was detected, indicating that there was no obvious publication bias in our study, which was also supported by Egger's test (*p* = 0.231 and *p* = 0.220 for incidence and survival, resp.) and Begg's test (*p* = 0.707 and *p* = 0.806 for incidence and survival, resp.).

## 4. Discussion

In this study, we analyzed the association of metformin use with the incidence and survival of EC using a meta-analysis to obtain a powerful conclusion. To the best of our knowledge, this is the first meta-analysis providing comprehensive insights into the effects of metformin use on the incidence and survival of EC. In the pooled analysis of five studies which evaluated the association of metformin use with the incidence of EC, we found that metformin use was associated with a 13% reduction in EC risk among patients with diabetes (RR = 0.87, 95% CI: 0.80–0.95; *p* = 0.006). In the pooled analysis of six retrospective cohort studies which investigated the effect of metformin use on the survival of EC patients, we found that, relative to nonuse, metformin use significantly improved the survival of EC patients (HR = 0.63, 95% CI: 0.45–0.87; *p* = 0.006).

Metformin, a biguanide, has become the most widely used antihyperglycemic drug. This drug is characterized by a broad spectrum of pleiotropic effects and good tolerability by patients. It may inhibit the growth and proliferation of gynecological cancer cells, such as those in breast cancer [30], ovary cancer [31], and cervical cancer [32]. In the past decade, many epidemiological studies showed the association between metformin use and the reduced risk and improved survival of patients with several types of cancers, including gynecological cancers. The beneficial effects of metformin use on EC may depend on common anticancer mechanisms present in other gynecological cancers [33] and even all tumors [34], but the exact molecular mechanism has not yet been fully elucidated.

The most frequently proposed hypotheses are the indirect effects of reducing levels of insulin and even insulin-like growth factor 1 (IGF-1), which is closely related to insulin signaling and exerts direct effects on the activation of cellular pathways of tumor cells. Metformin use reduced the blood glucose levels by the inhibition of gluconeogenesis in the liver [35], resulting in lowered circulating insulin levels. Epidemiological studies suggested that a high insulin level was associated with increased occurrence and mortality of cancers [36–38]. IGF-1 also plays a role in the occurrence and development of tumors [39], and the level of IGF-1 is significantly higher in EC compared with that in the normal endometrium [40]. Insulin/IGF-1 signaling is initiated by its binding to transmembrane receptors, which results in activation of tyrosine kinase activity. As with the activation of tyrosine kinase activity, adaptor proteins, such as the P85, are subsequently activated. P85 is the regulatory subunit of phosphatidylinositol 3-kinase (PI3-K) [41]. Insulin/IGF-1/PI3-K pathway plays a role in the carcinogenesis via mTOR, which is a key effector of PI3-K. In up to 80% of human cancers, mTOR is aberrantly activated [42]. Moreover, metformin use can also exert its direct antitumorigenic effects by activating AMPK, which participates in cellular proliferation and metabolism, including the inhibition of mTOR pathway [43]. mTOR plays a key role in cell growth and proliferation. Additionally, it participates in the formation of two protein complexes: mTOR complexes 1 and 2 (mTORC1/2). mTORC1 signaling is switched on by several oncogenic signaling pathways and is hyperactive in the majority of cancers [44].

The current meta-analysis has several strengths. First, the major strength of this study is that we comprehensively assessed the effects of metformin use on EC, using incidence and survival as the primary outcomes. Second, no obvious heterogeneity was detected in the evaluation of incidence of EC (*I*^2^ = 0.0%). Moreover, in the subgroup analyses of survival, the values of *I*^2^ were all less than 25%. Third, the sensitivity analysis did not show that a single study influenced the pooled results, and no publication bias was detected. Given these characteristics, this meta-analysis can be considered the most comprehensive study of metformin effects on EC, thus far.

However, several limitations in our study should be acknowledged. First, two of the eligible studies included in this meta-analysis did not report adjustments and the adjustments of the other nine studies were either inconsistent or incomplete. Many other confounders, such as cumulative dose, use of concomitant medications, and time-related bias, were not controlled. These aspects would be important to provide a more in-depth understanding of the nature of metformin use [45]. Second, the population of this study was based on Western and Asian populations. The lack of data from South America and Africa could possibly limit the generalizability of our conclusions. Therefore, it is necessary to verify the results in these areas. Moreover, this study was restricted to publications in English, which might also introduce publication bias. Finally, even though we found an obvious association between metformin use and the survival of patients with EC, current studies are unable to provide a conclusive result, because the sample size included in our meta-analysis is not sufficiently large. Thus, more prospective studies with large sample sizes are warranted.

## 5. Conclusions

In summary, our meta-analysis of observational studies demonstrated that metformin use reduces the incidence of EC among patients with diabetes and improves the overall survival of patients with EC. Our study results suggest that metformin may be a potential alternative treatment for patients with diabetes at high risk of EC and patients with EC and concomitant diabetes. However, accounting for the limitations of observational studies and the other limitations mentioned above, causality cannot be established yet. Further studies with large sample sizes, especially blind, randomized controlled clinical trials, are needed to confirm these results.

## Figures and Tables

**Table 1:** Characteristics of the included studies of metformin use and incidence of endometrial cancer.

Study (year)	Region	Study design	Date source	Cases/total subjects	Reference group	Time period	RR (95% CI)	Adjustment variables
Becker et al. 2013 [22]	UK	Case-control study	UK-based General Practice Research Database (GPRD)	291/1746	Nonmetformin	1995–2012	0.88 (0.58, 1.32)	BMI, smoking, and diabetes duration
Luo et al. 2014 [23]	USA	Retrospective cohort study	Women's Health Initiative	71/4247	Nonmetformin	2005–2010	0.97 (0.60, 1.58)	BMI
Ko et al. 2015 [24]	USA	Retrospective cohort study	Truven Health Analytics' MarketScan® and Medicare supplemental databases	NR/272411	Nonmetformin	2000–2011	0.89 (0.68, 1.17)	Age, Charlson index, PCOS, endometrial hyperplasia, obesity, combination oral contraceptive use, and ultrasound
Tseng 2015 [18]	Taiwan	Retrospective cohort study	National Health Insurance database of Taiwan	2885/478921	Nonmetformin	1996–2009	0.842 (0.761, 0.931)	Age, hypertension, COPD, stroke, nephropathy, ischemic heart disease, peripheral arterial disease, eye disease, obesity, dyslipidemia, urinary tract disease, other cancers, and other drugs
Franchi et al. 2016 [19]	Italy	Nested case-control study	The healthcare utilization databases of Lombardy	376/7485	Nonmetformin	1997–2012	1.07 (0.82, 1.41)	BMI

RR, relative risk; 95% CI, 95% confidence interval; NR, not reported; Nonmetformin, patients treated with other hypoglycemic drugs but not metformin.

**Table 2:** Characteristics of the included studies of metformin use and survival of endometrial cancer.

Study (year)	Region	Study design	Date source	Sample size	Stage	Reference group	Time period	HR (95% CI)	Adjustment variables
Ko et al. 2014 [20]	USA	Retrospective cohort study	NCI and NCCN designated academic institutions	363	All	Nonmetformin	2005–2010	0.43 (0.24, 0.77)	Age, stage, grade, histology, and adjuvant treatment
Nevadunsky et al. 2014 [25]	USA	Retrospective cohort study	Montefiore Medical Center (MMC)/Albert Einstein College of Medicine	985	All	Nonmetformin	1999–2009	0.54 (0.30, 0.97)	Age, clinical stage, grade, chemotherapy treatment, radiation treatment, and presence of hyperlipidemia
Lemańska et al. 2015 [26]	Poland	Retrospective cohort study	Department of Gynecologic Oncology of Poznan University of Medical Sciences	107	All	Nonmetformin	2002–2010	1.08 (0.46, 2.56)	NR
Ezewuiro et al. 2016 [27]	USA	Retrospective cohort study	The University of Chicago Medical Center (UCMC)	58	III, IV, and recurrence	Nonmetformin	1992–2011	0.42 (0.23, 0.78)	Study site, stage (III versus IV/recurrent), and age at chemotherapy
Seebacher et al. 2016 [28]	Austria	Retrospective cohort study	Department of Gynaecology and Gynaecological Oncology of the Medical University of Vienna	465	All	Nonmetformin	1995–2001	0.90 (0.69, 1.20)	NR
Al Hilli et al. 2016 [21]	USA	Retrospective cohort study	Patient database of Mayo Clinic, Rochester, Minnesota	138	All	Nonmetformin	1999–2008	0.61 (0.30, 1.23)	Propensity score

HR, hazard ratio; 95% CI, 95% confidence interval; NR, not reported; Nonmetformin, patients treated with other hypoglycemic drugs but not metformin.
